# Bat STING drives IFN-beta production in anti-RNA virus innate immune response

**DOI:** 10.3389/fmicb.2023.1232314

**Published:** 2023-09-08

**Authors:** Feiyu Fu, Qi Shao, Jianjian Zhang, Jie Wang, Zhaofei Wang, Jingjiao Ma, Yaxian Yan, Jianhe Sun, Yuqiang Cheng

**Affiliations:** Shanghai Key Laboratory of Veterinary Biotechnology, Key Laboratory of Urban Agriculture (South), Ministry of Agriculture, School of Agriculture and Biology, Shanghai Jiao Tong University, Shanghai, China

**Keywords:** bat, STING, IFN, innate immunity, RNA virus

## Abstract

The ability of stimulator of interferon genes (STING) to activate interferon (IFN) responses during RNA virus infection has been demonstrated in different mammalian cells. Despite being the host of numerous RNA viruses, the role of STING in bats during RNA virus infection has not been elucidated. In this study, we identified and cloned the STING gene of the Brazilian free-tailed bat *Tadarida brasiliensis* (*T. brasiliensis*) and tested its ability to induce IFN-β by overexpressing and knocking down bat STING (BatSTING) in *T. brasiliensis* 1 lung (TB1 Lu) cells. In addition, we used green fluorescent protein (GFP)-labeled vesicular stomatitis virus (VSV) VSV-GFP as a model to detect the antiviral activity of BatSTING. The results showed that overexpression of STING in TB1 Lu cells stimulated by cGAS significantly inhibited RNA virus replication, and the antiviral activities were associated with its ability to regulate basal expression of IFN-β and some IFN stimulated genes (ISGs). We also found that BatSTING was able to be activated after stimulation by diverse RNA viruses. The results of TB1 Lu cells with STING deficiency showed that knockdown of BatSTING severely hindered the IFN-β response triggered by VSV-GFP. Based on this, we confirm that BatSTING is required to induce IFN-β expression during RNA virus infection. In conclusion, our experimental data clearly show that STING in bat hosts plays an irreplaceable role in mediating IFN-β responses and anti-RNA virus infection.

## Introduction

Categorized as Chiroptera, bats account for 1,423 of more than 6,400 known mammal species, making them the most diverse and geographically widespread mammal after rodents (Sarkis et al., 2018; Clayton and Munir, 2020; Irving et al., 2021). Bats play a crucial role in the ecosystem by serving as pollinators, seed dispersers, and insect controllers. Recently, however, bats have been of increasing interest as reservoirs for numerous emerging zoonotic viruses (Banerjee et al., 2017; Arnold et al., 2018). Extensive laboratory studies and field observations have revealed that bats can carry numerous viruses, such as henipaviruses (Hendra and Nipah), coronaviruses (SARS-CoV), rhabdoviruses (Rabies and Lyssavirus), and filoviruses (Ebola and Marburg), but hardly show any signs of disease (Cowled et al., 2012; Baker et al., 2013; Zhou et al., 2016). Bats possess unique characteristics that set them apart from other mammals, potentially explaining their ability to host these viruses without experiencing clinical symptoms. Bats are the only true flying mammals, which allows them to carry viruses to distant areas (Han et al., 2015; Clayton and Munir, 2020). Their long lifespan relative to their size increases the potential for virus dispersal, and the swarming behavior of many bat species may facilitate the rapid spread of pathogens between bats and other species (Allen et al., 2009; Bolatti et al., 2020). For example, the Brazilian free-tailed bat *Tadarida brasiliensis* (*T. brasiliensis*), which roosts in some of the largest mammalian aggregations on Earth, is the most abundant migratory and cosmopolitan bat species in the New World (Bolatti et al., 2020).

In addition to various life history traits, bats have evolved unique immune systems that are particularly important for maintaining a harmonious virus-host relationship. The innate immune system recognizes microbial pathogens and activates intracellular and intercellular signaling pathways to combat infections (Anwar et al., 2021). Bats remain asymptomatic after viral infection, depending in large part on the effective control of virus replication by the host’s innate immune defense system (Tarigan et al., 2020). The innate immune response in the host is the first line of defense against pathogens, with the interferon (IFN) system playing a central role in this response (Franz et al., 2018; Subudhi et al., 2019). IFNs are a group of cytokines that are secreted by host cells following infection with pathogens (Clayton and Munir, 2020). The process of IFN induction and signaling after viral infections in non-bat mammals has been extensively discussed in many places. Briefly, IFN induction is stimulated when pathogen-associated molecular patterns (PAMPs) are recognized by cellular pattern recognition receptors (PRRs) (Maringer and Fernandez-Sesma, 2014; Pavlovich et al., 2020). Once release into the extracellular space, IFN signals by binding to specific receptors on both infected and uninfected cells, triggering various pathways to prevent intracellular replication and growth of pathogens, inducing an antiviral state in the host and impeding infection of peripheral cells (He et al., 2014; Zhou et al., 2016).

The activation of the IFN pathway involves different classes of PRRs: the toll-like receptor (TLR) family, the retinoic acid-inducible gene-I (RIG-I)-like receptors (RLRs), the nucleotide-binding oligomerization domain (NOD)-like receptors (NLRs), and cytosolic DNA sensors (Kumagai and Akira, 2010; Chen et al., 2017). In the context of viral infection, the most typical PAMPs are the viral genome itself or the viral nucleic acids generated during infection, such as single- or double-stranded RNA transcripts or DNA (Domizio et al., 2022). The role of different PRRs is related to the nature of the viral nucleic acids they sense. The TLRs and RLRs primarily react to viral RNA, while the DNA sensors primarily defend against viral DNA (Clayton and Munir, 2020). Among numerous DNA receptors, cyclic GMP-AMP synthase (cGAS)-stimulator of interferon genes (STING) (GAS-STING) axis has been identified as the primary innate immune pathway responsible for recognizing exogenous and endogenous cell membrane double-stranded DNA (dsDNA) (Ishikawa and Barber, 2008; Civril et al., 2013; Hopfner and Hornung, 2020).

The canonical cGAS-STING signaling cascade initiates with the detection of pathogenic or mislocated self-DNA in the cytoplasm, including nuclear DNA (nDNA) and mitochondrial DNA (mtDNA) (Anwar et al., 2021). Once the DNA is identified, the enzymatic activity of cGAS is activated, leading to the synthesis of 2′-3′cGAMP using GTP and ATP as substrates (Ishikawa and Barber, 2008). Then, 2′-3′cGAMP works as a second messenger, activating the adapter protein interferon gene stimulator (STING; also known as MITA, ERIS and MPYS). This activation induces conformational changes in STING, causing its translocation from the endoplasmic reticulum (ER) to the ER-Golgi intermediate compartment (ERGIC) and the trans-Golgi network (TGN) (Ma and Damania, 2016). In this process, STING recruits and activates TANK-binding kinase 1 (TBK1) and inhibitor nuclear factor kappa B (IκB) kinase (IκK). The latter two induce phosphorylation of interferon regulatory factor 3 (IRF-3) and IκB alpha (IκBα), respectively (Fan et al., 2022). Phosphorylated IRF-3 dimerizes and subsequently translocates to the nucleus, triggering the expression of type I IFN (IFN-I) and further production of IFN-stimulated genes (ISGs) (Amurri et al., 2023). Activated nuclear factor kappa B (NF-κB) translocates to the nucleus and drives the transcription of genes encoding inflammatory cytokines (Anwar et al., 2021).

With the exception of playing a key role in the innate immune response against DNA viruses, there is growing evidence for an significant contribution of the cGAS-STING axis in the control of RNA virus infection (Amurri et al., 2023). It is well known that RNA virus infection also leads to cytoplasmic DNA production due to intracellular damage. Certain RNA viruses, like dengue virus (DENV) (Chatel-Chaix et al., 2016), have the ability to cause mitochondrial stress in infected cells through corresponding mechanisms, leading to the rupture of mitochondrial membrane and the release of mtDNA into the cytoplasm. The released dsDNA is then detected by cGAS and results in activation of STING and induction of downstream IFN-I, thus conferring host resistance to RNA viruses. In addition, some RNA viruses such as flaviviruses (Amurri et al., 2023) can indirectly activate the cGAS-STING axis along the RLRs pathway although they do not show any DNA intermediate step in their replication cycle. The cGAS-STING pathway has been reported to strongly evoke IFN responses and effectively inhibit infection by several other RNA viruses, such as chikungunya virus (CHIKV) (Webb et al., 2020), encephalomyocarditis virus (EMCV) (Cui et al., 2020), hepatitis C virus (HCV) (Yi et al., 2016), murine norovirus (MNV) (Yu et al., 2021), Nipah virus (NiV) (Iampietro et al., 2021), and Zika virus (ZIKV) (Liu and Cherry, 2019). Cells lacking STING show defective IFN activation in response to infection by some RNA viruses such as vesicular stomatitis virus (VSV) and Sendai virus (SeV) (Ishikawa et al., 2009). On the other hand, as a key adapter protein in the cGAS-STING signaling pathway, multiple RNA viruses from different viral families have developed direct or indirect strategies to antagonize STING through different structural and nonstructural proteins (Ishikawa et al., 2009; Aguirre et al., 2012). The emerging SARS-CoV-2 in recent years has multiple mechanisms to suppress the antiviral activity of cGAS-STING at the STING level have been highlighted (Rui et al., 2021). Influenza A virus (IAV) hemagglutinin (HA) can also block STING dimerization and TBK1 phosphorylation, thereby reducing STING-dependent IFN-I production (Holm et al., 2016). These phenomena reveal that RNA viruses must develop mechanisms to escape this immune signaling pathway, highlighting the importance of the cGAS-STING pathway in RNA virus infection.

For the past few years, the understanding of signaling interactions between RNA viruses and cGAS-STING has advanced significantly. The vital role of the cGAS-STING axis in the innate immune response to RNA viruses in humans (Domizio et al., 2022), mice (Yu et al., 2021), pigs (Xu et al., 2021) and other mammals has been described. And homologs of STING have been confirmed in several bat species of *Myotis davidii*, *Rhinolophus sinicus* and *Pteropus Alecto* (Xie et al., 2018). Studies of them have shown that STING-dependent IFN activation is weakened in bats (Xie et al., 2018). However, it is uncertain whether this IFN activation defect in STING is common to all bats or whether it is due to phylogenetic differences and deviations in gene expression patterns among different bat species. Indeed, due to the diversity of bat species and the limited availability of bat cell models, information on the role of bat immune responses in controlling viral infections is still lacking. The specific mechanism of cGAS-STING signaling involved in IFN production against RNA viruses in bats urgently needs further investigation.

In the present study, we cloned and molecularly characterized the STING gene from the Brazilian free-tailed bat *T. brasiliensis*. Using human cGAS as an agonist, we investigated the potential involvement of STING-mediated innate immune response to RNA viruses in bat cells. We found that the expression level of BatSTING was significantly increased after bat cells were infected with three RNA viruses, NDV-GFP, VSV-GFP, and AIV. In addition, stimulated by cGAS, BatSTING was able to induce the expression of IFN-β as well as some ISGs and pro-inflammatory cytokines in bat cells and conferred a robust state of cellular resistance to RNA virus infection. Further results of RNA interference (RNAi) experiments suggest that BatSTING is necessary for the induction of IFN response after RNA virus VSV-GFP infection.

## Materials and methods

### Cell lines and viruses

*T. brasiliensis* 1 lung (TB1 Lu) is a lung epithelial cell line derived from the Brazilian free-tailed bats. The TB1 Lu cells were obtained from ATCC and cultured in high-glucose complete Dulbecco’s Modified Eagle’s Medium (DMEM; Gibco, United States) supplemented with 10% fetal bovine serum (FBS; Nulen, Shanghai, China) and 1% penicillin-streptomycin (Gibco). The cells were cultured in a 37°C, 5% carbon dioxide incubator. Low virulent strain LaSota of Newcastle disease virus (NDV) tagged Green fluorescent protein (GFP) named NDV-GFP, and GFP tagged vesicular stomatitis virus (VSV) VSV-GFP were stored in our laboratory. The A/Chicken/Shanghai/010/2008 (H9N2) virus was isolated from chicken in Shanghai, China, in 2008 and identified as H9N2 avian influenza virus (AIV). These three viruses were propagated and purified as described in our previous study (Cheng et al., 2015).

### PCR amplification and bioinformatics analysis of BatSTING

According to the predicted sequence of *Molossus molossus* STING sequence (XM_036241931.1) obtained from the National Center for Biotechnology Information (NCBI), the primers BatSTING-F and BatSTING-R (Table 1), were designed and used to amplify potential BatSTING cDNA via reverse transcription-polymerase chain reaction (RT-PCR) on total RNA extracted from TB1 Lu cells. The PCR products were ligated into a pTOPO-Blunt vector (Aidlab Biotech, Beijing, China) for sequencing, and the positive colonies were sent to the Beijing Tsingke Biotech Co., Ltd. (Beijing, China) for sequencing. Conserved domains in the amino acid sequence of BatSTING were analyzed using the simple modular architecture research tool (SMART) program.^1^ The amino acid sequence of BatSTING was aligned with STING from other species such as humans, pigs, mice, chickens and ducks using Clustal W and edited with ESPript 3.0.^2^ Sequence homology and a phylogenetic analysis of amino acid sequences were conducted using DNASTAR software. Homology modeling for BatSTING was built by the online protein-modeling server SWISS-MODEL.^3^

**Table 1:** PCR primers used in this study.

Target gene	Purpose	Name	Sequence of oligonucleotide (5′-3′)
BatIFN-β	RT-qPCR	qbatIFN-β F	GCACCGGCTGGAATGAGACCA
		qbatIFN-β R	GTCCAGGCATTGGCTGT
BatMx-1	RT-qPCR	qbatMx-1 F	GGAGGGTCAGCTCCCCTCA
		qbatMx-1 R	GCCATGCTCAGCGCCTCT
BatPKR	RT-qPCR	qbatPKR F	ACCTTCTGTGAGCAGTGTTAG
		qbatPKR R	CTGTGGTCCATAGTGTACTCATT
BatOAS1	RT-qPCR	qbatOAS1 F	ATCTGCAGTTTCCTGAAGGAG
		qbatOAS1 R	GCTGAGGAAGCGACGAGGTC
BatTNF-α	RT-qPCR	qbatTNF-α F	TGACAAGCCTGTTGCCCATGT
		qbatTNF-α R	TGAAGAGGACCTGGGAGTAGA
BatIL-6	qRT-PCR	qBatIL-6 F	CTACTGCTTTCCCTACCC
		qBatIL-6 R	TCCTTGCTGTTTTTACACG
BatIL-1β	qRT-PCR	qBatIL-1β F	CTCCGGGACATAAACCAGAAG
		qBatIL-1β R	CTGGGATCTTGTCATCGTTCTC
Batβ-actin	RT-qPCR	qbatβ-actin F	CCATCCTGCGTCTGGACCTGG
		qbatβ-actin R	GTGGCCATCTCCTGCTCGAAG
STING	RT-qPCR	qbatSTING F	ATGGGCTGGCATGGTCATTT
		qbatSTING R	GCTTCCTGCGCTTCGTAGTA
	To obtain sequence	BatSTING F	ATGCCCCACTCCAGCTTGCAC
		BatSTING R	TCAGAAGATATCTGTGCGGAG
	Construction of BatSTING	pc*DNA*3.1-Flag EcoR I	TAGTCCAGTGTGGTGGAATTC
			ATGAGGATTGCTGAGGAG
		pc*DNA*3.1-Flag Xho I	GTCGTCCTTGTAGTCCTCGAG
			GAAGATATCTGTGCGGAGCGG
	Construct truncated forms of BatSTING	BatSTING d1-17 aa F	AGTGTGGTGGAATTCGCCAAG
			AAGGCAGCCGTTGT
		BatSTING d1-17 aa R	GAATTCCACCACACTGGACTA
			GTGGATCCGAGCTC
		BatSTING d1-32 aa F	AGTGTGGTGGAATTCTTTTGG
			GGTTTGAGTGAACC
		BatSTING d1-32 aa R	GAATTCCACCACACTGGACTA
			GTGGATCCGAGCTC
		BatSTING d33-43 aa F	GCCTGCCTGGCAGCCCTCCAG
			TGGCTGGTGCTCCA
		BatSTING d33-43 aa R	GGCTGCCAGGCAGGCACTCA
			GCAGGACAACGGCTG
		BatSTING d146-334 aa F	CCAGCTGAGGTGTCTAAGGAG
			GAGGTTACTGTGGG
		BatSTING d146-334 aa R	AGACACCTCAGCTGGGGCCG
			GGACCTGGAGGTTCA
		BatSTING d335-376 aa F	CTGCAGCAGGAGGAACTCGAG
			GACTACAAGGACGA
		BatSTING d335-376 aa R	TTCCTCCTGCTGCAGGTGCCT
			GAGAATCTCCTGTG
		BatSTING H358S F	GAGGAGTCTGAGCTCCTCATC
			AGTGAAATGGATCC
		BatSTING H358S R	GAGCTCAGACTCCTCGGGCAG
			CGTGGAAGAGCCAG

### Plasmids construction and transfection

Using the Hieff Clone^®^ Universal One-Step Cloning Kit (Yeasen, Shanghai, China), the full-length BatSTING was inserted into the EcoR I and Xho I sites of the pcDNA3.1 Flag expression vector to construct the BatSTING plasmid with the Flag tag (pcDNA3.1-BatSTING-Flag). The bat IFN-β (batIFN-β) promoter luciferase reporter plasmids pGL-batIFN-β-Luc was constructed from TB1 Lu genomic DNA using primers with *Nhe* I and *Bgl* II sites (IFN-β-P F and IFN-β-P R) to amplify −500 to −1 of the batIFN-β promoter motif. The promoter fragment was inserted between *Nhe* I and *Bgl* II sites of the pGL3.0-basic luciferase reporter vector (Promega, Madison, WI). The truncated plasmids of BatSTING, including BatSTING-d1-17aa, BatSTING-d1-32aa, BatSTING-d33-43aa, BatSTING-d146-334aa, BatSTING-d335-376aa and BatSTING-H358S, were constructed using a modified homologous recombination method and the primers listed in Table 1. The Trelief^™^ 5α Chemically Competent Cell (Tsingke Biological Technology) was used for plasmid transformation. The plasmids were transfected with Nulen PlusTrans^™^ Transfection Reagent (Nulen, Shanghai, China) according to the manufacture’s protocol.

### Viral infection and quantification

The titers of the three viruses NDV-GFP, VSV-GFP and AIV were determined by the 50% tissue culture infective dose (TCID50) calculated according to the method of Reed–Muench. Antiviral activity was assessed by transfecting TB1 Lu cells with overexpressing plasmid or empty vector plasmid. After 24 h, the transfected cells were washed twice with phosphate buffer saline (PBS) (Gibco) and infected with VSV-GFP at 1 multiplicity of infection (MOI), and fluorescence was measured 12 h and 24 h after infection through a fluorescence microscope. Quantification of mean fluorescence intensity was performed using ImageJ. The RNA from the cells, which was infected with 1 MOI of NDV-GFP or VSV-GFP or AIV at different time, was collected for reverse transcription quantitative real-time PCR (RT-qPCR).

### Western blot

The TB1 Lu cells were seeded in 12-well plates (NEST Biotechnology, Wuxi, China) at 1 × 10^6^/mL and then transfected with a total of empty plasmid or overexpression plasmid. At 24 h post-transfection, cells were washed twice with PBS (Gibco) and then lysed with Radioimmunoprecipitation assay (RIPA) buffer (Beyotime, Shanghai, China) supplemented with 1% phenylmethylsulfonyl fluoride (PMSF) (Yeasen). Lysates were centrifuged at 13,000 rpm for 10 min to obtain the supernatant. Then the lysates were eluted with a 5 × SDS-PAGE loading buffer (Yeasen) before boiling for 10 min. Proteins isolated from the cell lysates were separated via SDS-PAGE and analyzed by western blot. The antibodies included mouse anti-GFP (at 1:5000 dilution; ABclonal, Wuhan, China), mouse anti-Flag (at 1:5000 dilution; Nulen), rabbit anti-TBK1 (at 1:1000 dilution; Cell Signaling Technology), rabbit anti-phospho-TBK1 (at 1:1000 dilution; Cell Signaling Technology) and anti-β-tubulin (at 1:7000 dilution; Yeasen) overnight at 4°C. The membrane was washed 3 times for 5 min each with tris buffered saline and Tween-20 (TBST) (Sangon Biotech Co., Ltd., Shanghai, Beijing, China). Then, the secondary antibody goat anti-mouse IgG (at 1:10000 dilution; Nulen) or goat anti-rabbit IgG (at 1:1000 dilution; Cell Signaling Technology) was added for 1 h incubation at room temperature. Images were obtained with the Tanon 5200 imaging system (Tanon, Shanghai, China), as described in our previous study (Niu et al., 2019).

### Dual luciferase reporter assays

Activation of the bat IFN-β promoter was analyzed by IFN-β promoter activation reporter assay. TB1 Lu cells were inoculated in 24-well plates at a cell density of 5 × 10^5^/mL. Plasmids with optimized amount (0.14 μg/well reporter plasmid pGL-batIFN-β-Luc, 0.07 μg/well internal control Renilla luciferase (pRL-TK) along with the indicated plasmids) were transiently transfected into TB1 Lu cells. Then the cells were lysed 24 h after transfection, and luciferase activity was determined using a Dual-Luciferase Reporter Assay System kit (Promega, United States) according to the manufacturer’s instructions. The ratio of Firefly to Renilla luciferase signal was calculated and then normalized to the wells transfected with empty vector. All reporter assays were repeated at least three times.

### Reverse transcription quantitative real-time PCR

The total RNA was extracted from TB1 Lu cells using AG RNAex Pro Reagent (Accurate Biology, Hunan, China) and converted to cDNA with a cDNA synthesis kit (Vazyme). For each sample, 1 μg RNA was applied to RT-PCR and the reaction consisted of two steps: removal of genomic DNA and reverse transcription, using the following RT-PCR conditions: 42°C for 2 min, followed by 37°C for 15 min and 85°C for 5 s. Relative gene expression was determined by a ChamQTM SYBR^®^ qPCR Master Mix (Vazyme) on the Applied Biosystems machine (ABI 7500; Thermo Fisher Scientific) and was analyzed using the 2^−∆∆Ct^ method. When examining gene levels, β-actin was the internal reference and its Ct value was stable within a certain range across different experimental treatment conditions. Primer sequences for the genes are shown in Table 1.

### RNA interference

pGPU6/Neo was used to knockdown the BatSTING gene (GenePharma, Shanghai, China). Two RNAi plasmids were designed by the GenScript RNAi target finder^4^ and cloned into the shRNA expression vector pGPU6/Neo (Table 2) (GenePharma), yielding shSTING-1 and shSTING-2. shNC was used as a negative control plasmid encoding a hairpin siRNA sequence not present in human or bat genome databases. For silencing, TB1 Lu cells in 12-well plates were transfected with shRNA plasmids at 1 μg/mL using Nulen PlusTrans^™^ Transfection Reagent (Nulen) according to the manufacture’s protocol. After 24 h, cells were infected with VSV-GFP at 1 MOI for 24 h. The expression of each immune-related gene was detected by RT-qPCR.

**Table 2:** The sequences of shRNAs used in this study.

Target gene	Name	Sequence of oligonucleotide (5′-3′)
STING	shSTING-1	CATGGGCTGGCATGGTCATTT
	shSTING-2	CAGAACAACTGTCGCTTAATT
Negative control	shNC	GTTCTCCGAACGTGTCACGT

### Statistical analysis

Data were expressed as means ± standard deviations. GraphPad Prism 8.0 was utilized to graph the results. The two-tailed independent student’s *t*-test or one-way analysis of variance (ANOVA) was used to determine the statistically significance. (^*^p < 0.05, ^**^p < 0.01, ^***^p < 0.001, ^****^p < 0.0001).

## Results

### Cloning and sequence analysis of BatSTING

**Figure 1:**
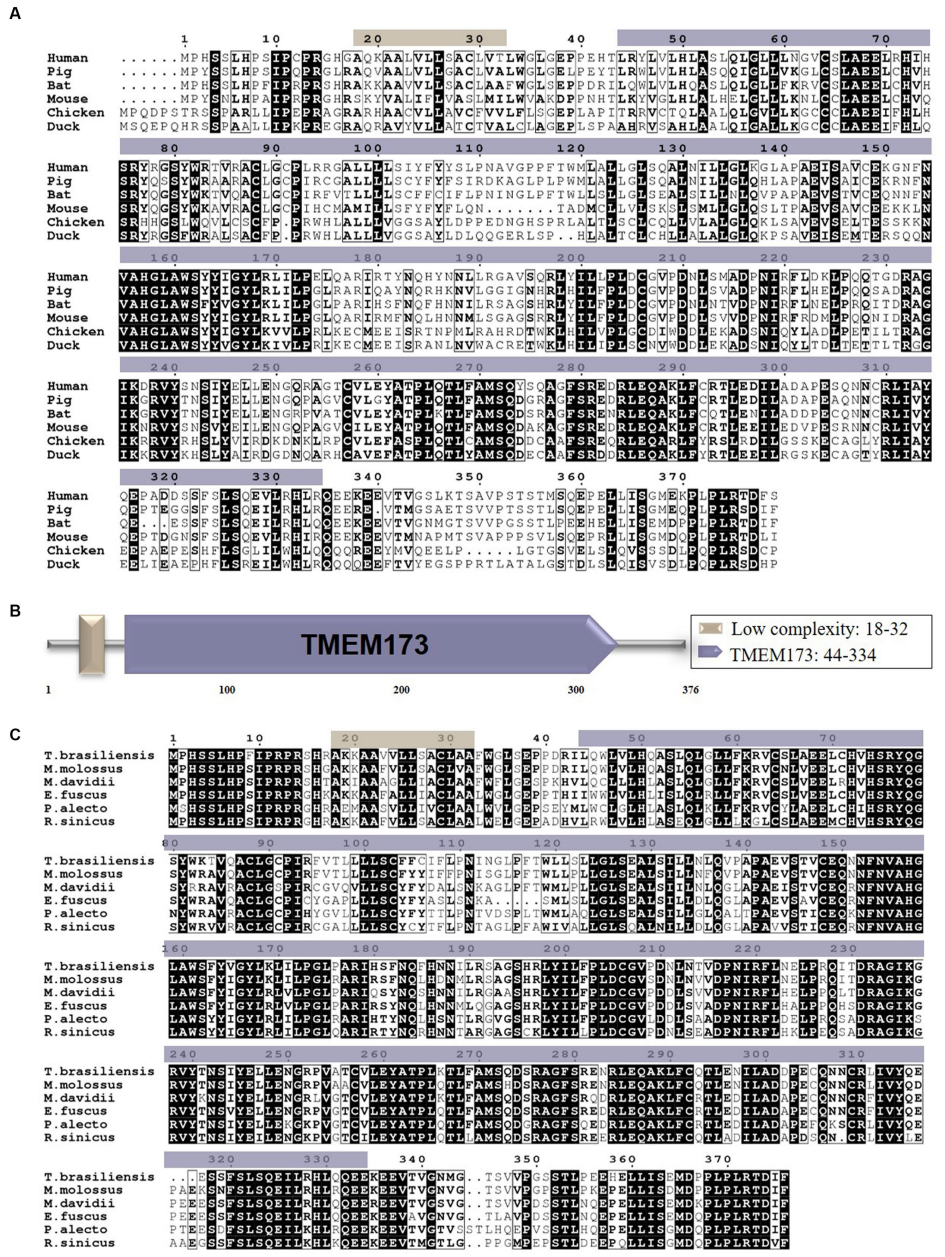
**(A)** The alignment of the deduced amino acid sequence of BatSTING with other animal STING proteins from the humans, pigs, mice, chickens and ducks was performed using the Clustal W program and edited with ESPript 3.0. The black shading indicates the identity of the amino acid, and the gray shading indicates similarity (50% threshold). **(B)** The prediction of protein domains of BatSTING. **(C)** The alignment of the amino acid sequence of *T. brasiliensis* STING with other deduced STING proteins from *M. molossus*, *M. davidii*, *E. fuscus*, *P. alecto* and *R. sinicus* was performed using the Clustal W program and edited with ESPript 3.0. The black shading indicates the identity of the amino acid, and the gray shading indicates similarity (50% threshold).

To explore the role of STING in the innate immune system of bats, we cloned the full-length BatSTING gene from cDNA using RT-PCR on total RNA extracted from TB1 Lu cells. The ORF region of BatSTING contains 1,131 bp, encoding 376 amino acid (aa) residues (Figure 1A). We used the SMART program to predict the secondary structure of BatSTING and to elucidate the molecular function of BatSTING. The prediction results show that BatSTING has a typical TMEM173 domain (44-334aa) and a low complexity (18-32aa) (Figure 1B). Multiple alignment results showed BatSTING amino acid sequences identities 72.3% to humans (NP_938023.1), 72.3% to pigs (NP_001136310.1), 65.3% to mice (NP_082537.1), to 41.6% chickens (XP_046783054.1) and 40.9% to ducks (XP_027311055.1). It was found that the sequence similarity of STING of these six species was low. We also compared the amino acid sequence of the cloned STING from *T. brasiliensis* with the STING sequences from several other bat species including *M. molossus*, *M. davidii*, *Eptesicus fuscus*, *P. Alecto* and *R. sinicus* (Figure 1C). Preliminary analysis shows that the amino acid sequence of STING is relatively conserved among different species of bats. Among them, *M. molossus* STING has the highest sequence similarity with *T. brasiliensis* STING at 78.8%. The sequence similarities between STING from *M. davidii*, *R. sinicus* and *P. Alecto* and *T. brasiliensis* are close, ranging from 63.8% to 68.4%. The lowest similarity with *T. brasiliensis* STING is that of *E. fuscus*, which is only 27.8%, indicating that although they belong to the same species, there may be large differences in the inherent functions of innate immune molecules between different bats.

### Phylogenetic tree analyses and the three-dimensional structure of BatSTING

**Figure 2:**
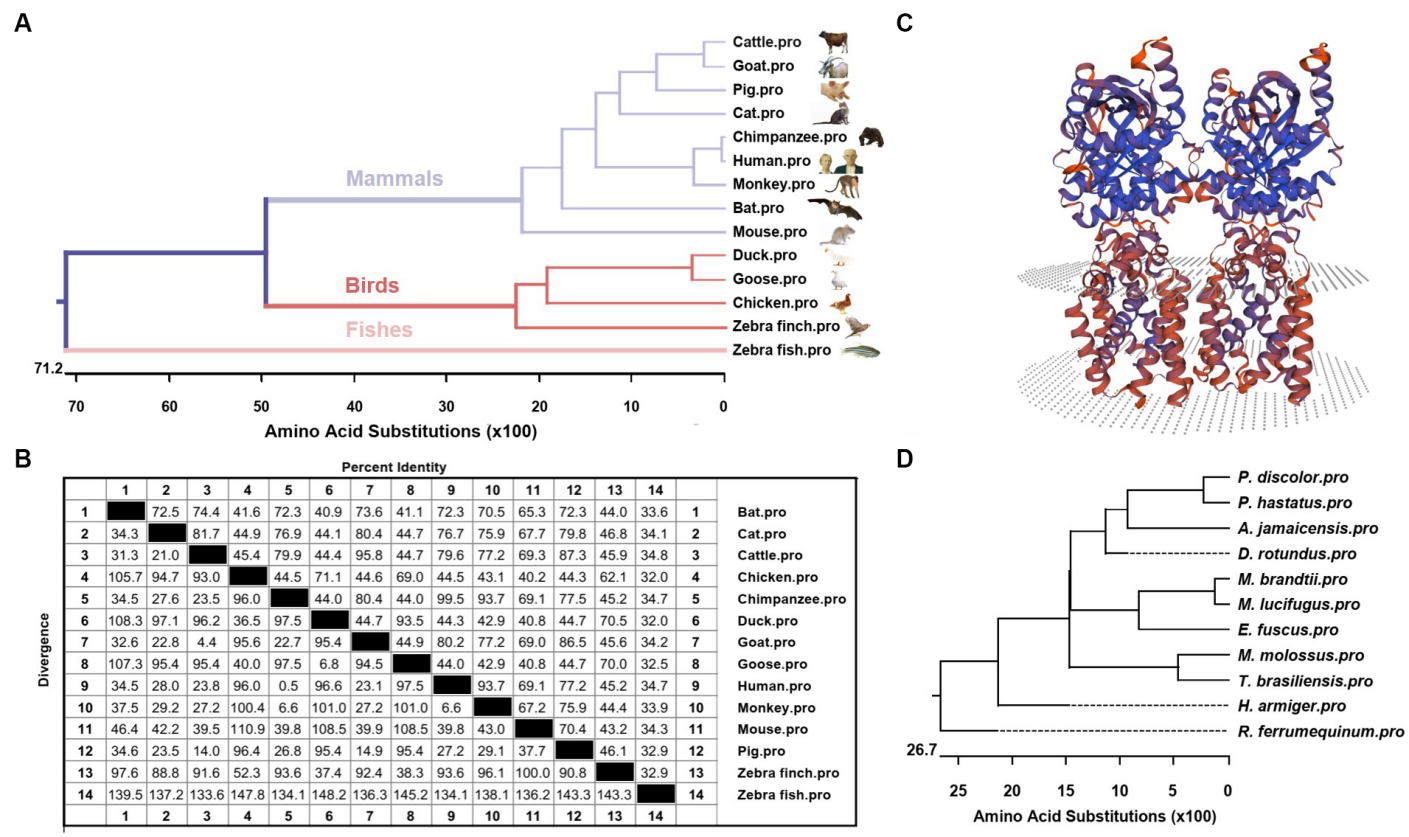
**(A)** Phylogenetic tree of the deduced amino acid sequence of BatSTING and other animal STING proteins. **(B)** The amino acid sequence homology of STING in different animals. **(C)** Three-dimensional structure of BatSTING predicted by SWISS-MODEL. **(D)** Phylogenetic tree of the deduced amino acid sequence of STING among different bat species.

Phylogenetic tree analysis was conducted to infer the kinship of STING among different species including mammals, birds and fishes. The analysis results showed that bats were grouped together with other mammals, including cattle, goats, pigs, cats, chimpanzees, humans, monkeys and mice. STING protein sequences from ducks, geese, chickens and zebra finches formed another subgroup, while zebrafish STING belonged to a separate subgroup (Figure 2A). To determine the homology between the amino acid sequences of STING from different species, the software MegAlign was used, and the results are presented in Figure 2B. In addition, to gain insight into the evolutionary relationships of STING within species, we conducted a phylogenetic analysis of STING from 11 different bat species (Figure 2D). The intraspecific phylogenetic tree consisted of three main branches, in which *T. brasiliensis* belonged to the same branch as the Pallas’s Mastiff Bat (*M. molossus*), the big brown bat (*E. fuscus*), the little brown bat (*Myotis lucifugus*), the Brandt’s bat (*Myotis brandtii*), the common vampire bat (*Desmodus rotundus*), Jamaican fruit-eating bat (*Artibeus jamaicensis*), greater spear-nosed bat (*Phyllostomus hastatus*), and the pale spear-nosed bat (*Phyllostomus discolor*) and are evolutionarily most closely related to *M. molossus*. The predicted three-dimensional structure of BatSTING is shown in Figure 2C.

### BatSTING can be highly upregulated after RNA virus stimulation

**Figure 3:**
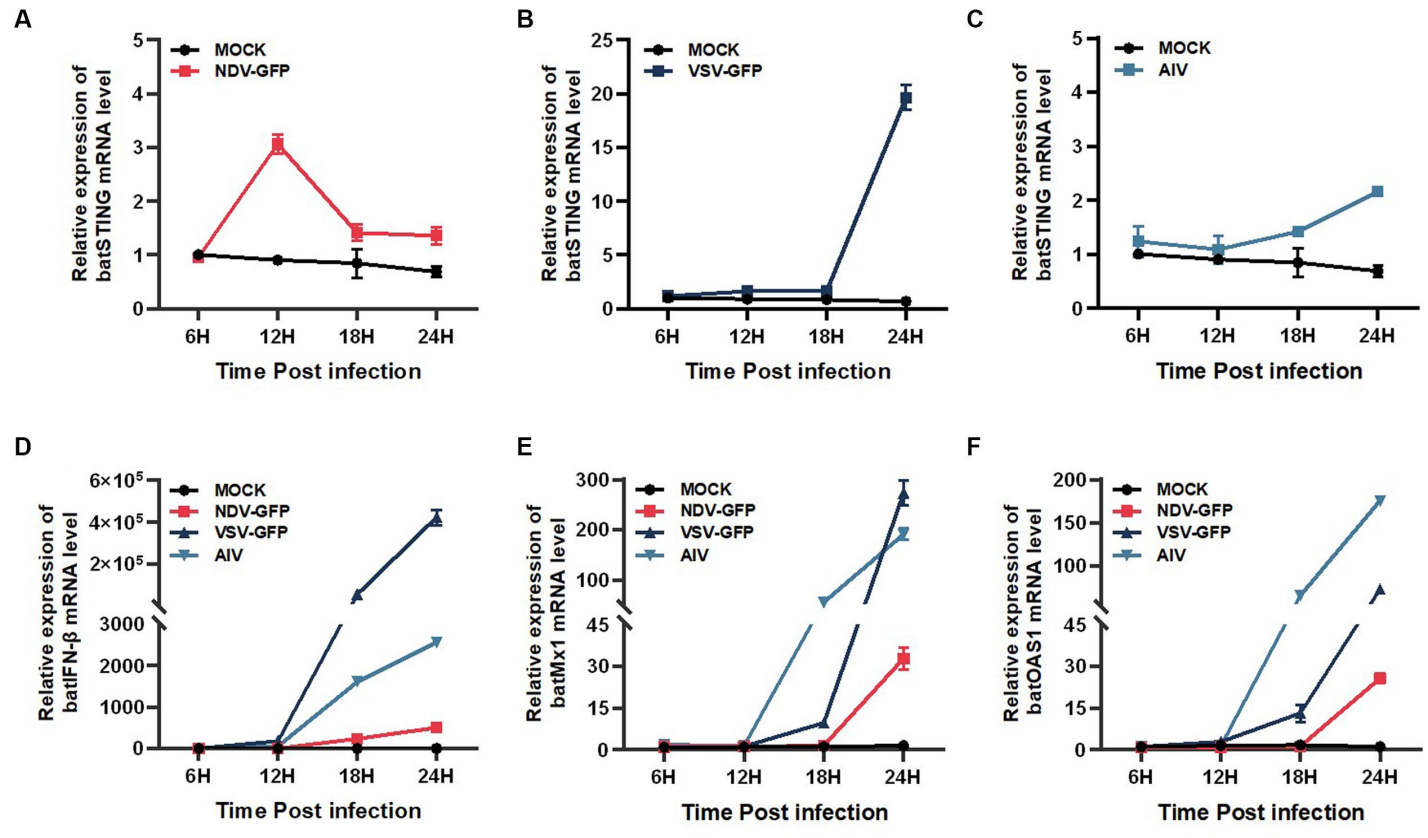
BatSTING can be highly upregulated after RNA Virus stimulation. The relative mRNA levels of BatSTING **(A–C)**, IFN-β **(D)** and ISGs (Mx-1 and OAS1) **(E,F)** were analyzed using RT-qPCR in TB1 Lu cells infected with NDV-GFP, VSV-GFP and AIV at 1 MOI. Error bars represent standard deviations.

To determine whether BatSTING can be induced in the presence of RNA virus infection, we analyzed the expression of BatSTING, IFN-β, and ISGs in TB1 Lu cells following infection with NDV-GFP, VSV-GFP and AIV using RT-qPCR. The results showed that the expression of BatSTING was significantly upregulated at the transcriptional level in all three RNA virus-infected TB1 Lu cells (Figures 3A–C). The mRNA levels of IFN-β (Figure 3D) and ISGs (Mx1 and OAS1) (Figures 3E,F) were also significantly upregulated. Cells resist the invasion of foreign viruses by regulating the expression of these genes.

### Activation of BatSTING suppresses VSV-GFP infection in bat cells

**Figure 4:**
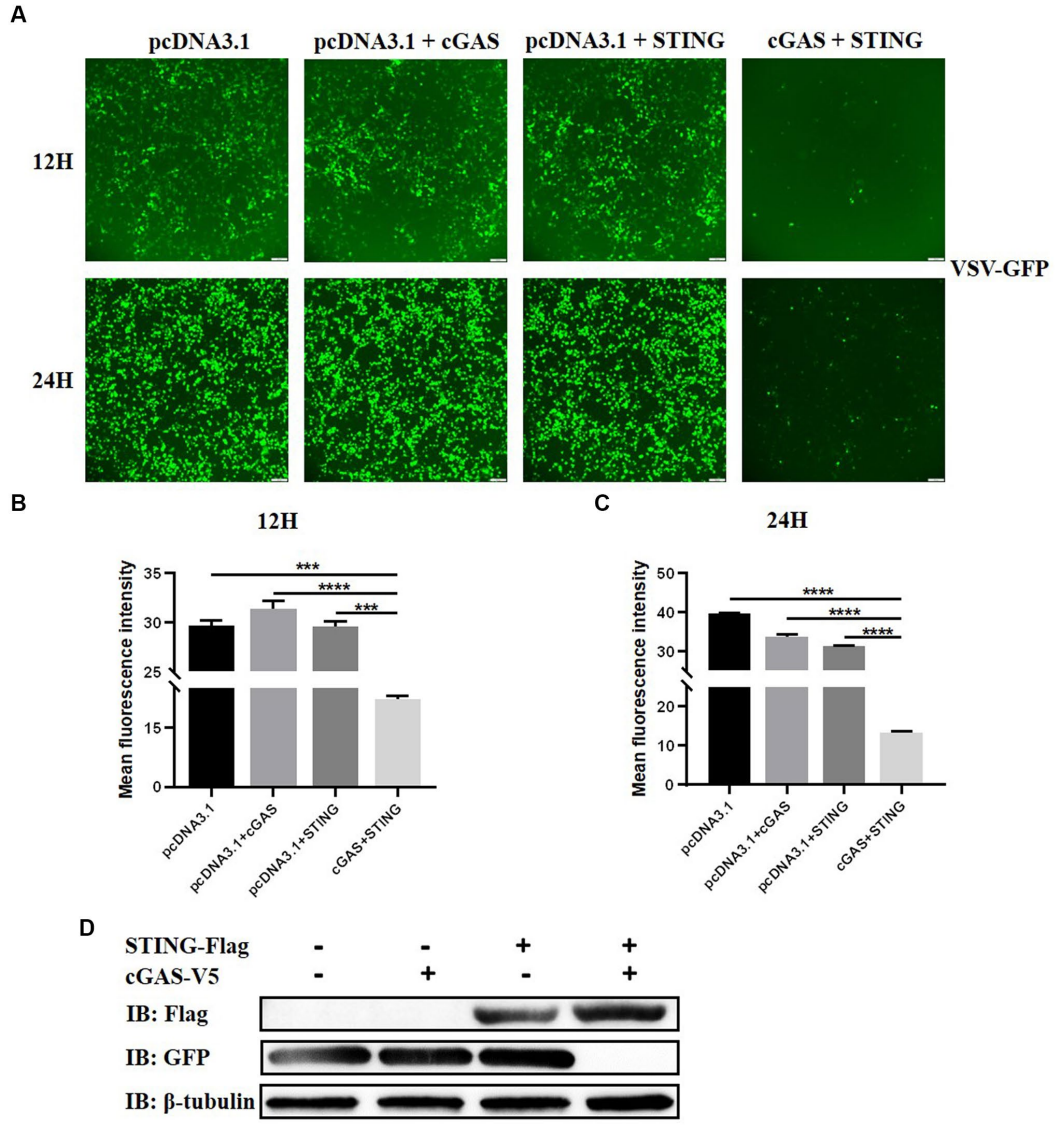
Activation of cGAS-STING pathway suppresses VSV-GFP infection in bat cells. **(A)** Viral fluorescence in TB1 Lu cells transfected with HucGAS, BatSTING or combination and infected with VSV-GFP at 1 MOI. **(B,C)** The mean fluorescence intensity of the virus after 12 h and 24 h infection with VSV-GFP in TB1 L cells co-overexpressing HucGAS and BatSTING. **(D)** The expression of STING-Flag and viral replications in TB1 Lu OE cells were also monitored by western blots. Data are expressed as the means ± SD of three independent experiments. Data are represented as mean ± SD. The signs “^***^” and “^****^” denote ^***^p < 0.001 and ^****^p < 0.0001, respectively.

To analyze the impact of STING on RNA virus replication in TB1 Lu cells, the gene overexpressing (OE) TB1 Lu cells were infected with VSV-GFP, and fluorescence was monitored under a fluorescence microscope. The mean fluorescence intensities of VSV-GFP reflects its replication circumstances in TB1 Lu cells. As expected, the viral fluorescence intensity in cGAS OE and STING OE cells was significantly lower than that in control TB1 Lu cells 12 h and 24 h after viral infection (Figures 4A–C). The replication circumstances of VSV-GFP were also monitored by western blot of the virus. The results showed a lower abundance of viral proteins in cGAS OE and STING OE cells compared to control cells (Figure 4D). All of these results indicate that the activation of STING in TB1 Lu cells could obviously inhibit RNA virus VSV-GFP viral replication.

### BatSTING regulates the expression levels of IFN-β and ISGs

**Figure 5:**
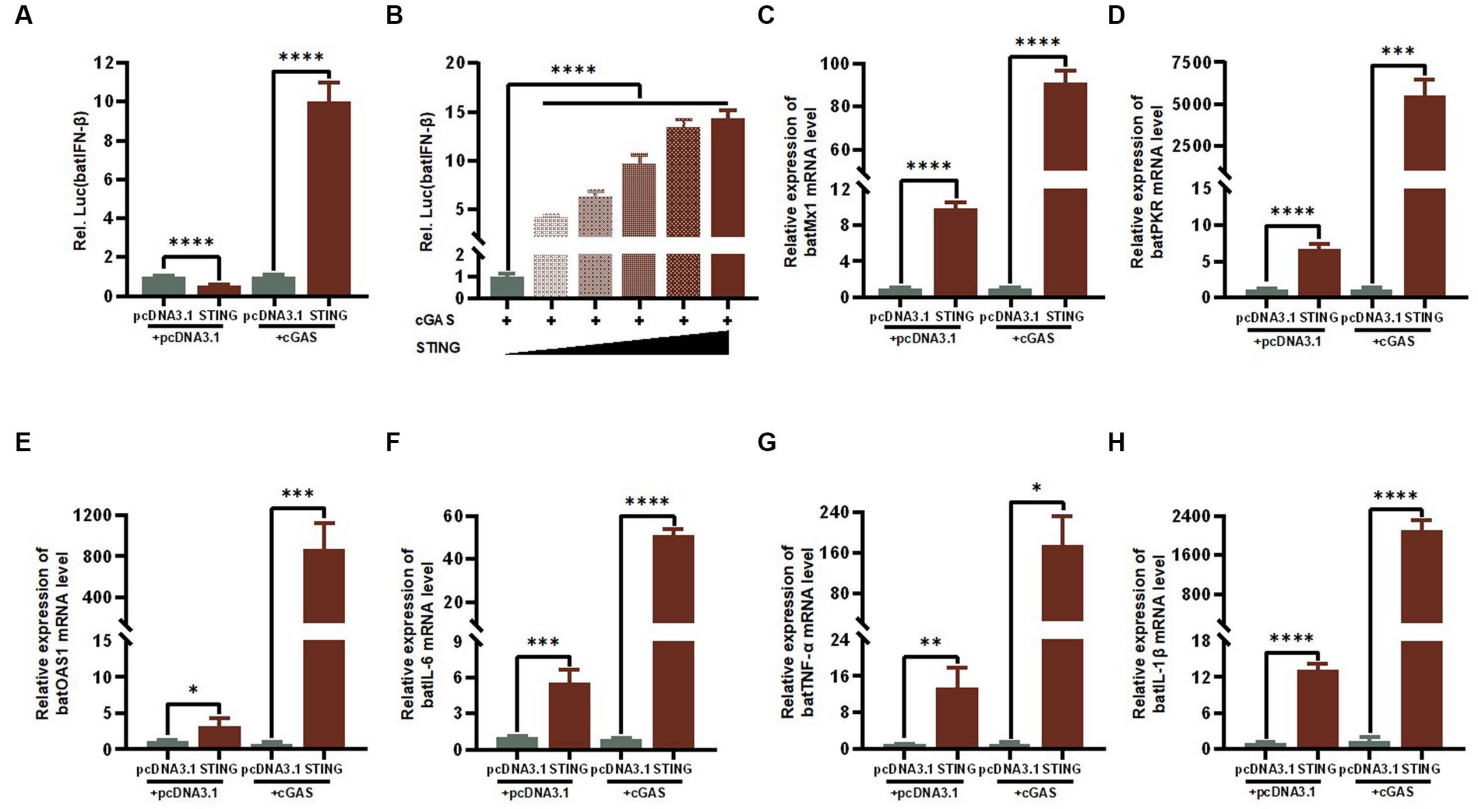
The cGAS-STING signaling axis regulates the expression levels of IFN-β and ISGs. **(A)** TB1 Lu cells grown in 24-well plates (2 × 10^5^ cells/well) were co-transfected with HucGAS, BatSTING plus pGL-batIFN-β-luc and pRL-TK. Luciferase activities were measured 24 h post-transfection. **(B)** BatSTING dose-independently induced IFN-β induction in HucGAS treated TB1 Lu cells. TB1 Lu cells grown in 12-well plates (1 × 10^6^ cells/well) were transfected with HucGAS, BatSTING or combination. Twenty-four hours post transfection, the expression of Mx1 **(C)**, PKR **(D)**, OAS1 **(E)**, IL-6 **(F)**, TNF-α **(G)** and IL-1β **(H)** genes in TB1 Lu cells were analyzed by RT-qPCR. ns, not significant; ^*^p < 0.1; ^**^p < 0.01; ^***^p < 0.001; ^****^p < 0.0001.

IFNs are one of the most important lines of defense in the innate immune response to inhibit viral replication. We therefore reasoned that the antiviral activity of STING is most likely mediated through the activation of IFN-I and its downstream series of ISGs. To examine the regulation of STING on key immune-related genes in the bat’s IFN-I signaling pathway, we transfected TB1 Lu cells with constructs that overexpressed cGAS and STING and empty vectors, individually or co-overexpressed, and examined batIFN-β activation with a luciferase reporter assay. The results showed that either single overexpression STING or common overexpression of cGAS and STING resulted in significant activation of the IFN-β promoter in TB1 Lu cells (Figure 5A), which was positively dose-dependent (Figure 5B). We then detected the mRNA expression levels of other immune-related genes using RT-qPCR. It was found that the stimulation of STING in TB1 Lu cells significantly increased the expression of ISGs (Mx1, PKR and OAS1) (Figures 5C–E) and pro-inflammatory cytokines IL-6, TNF-α and IL-1β (Figures 5F–H).

### Knockdown of BatSTING blocks IFN-β induction during RNA virus infection

**Figure 6:**
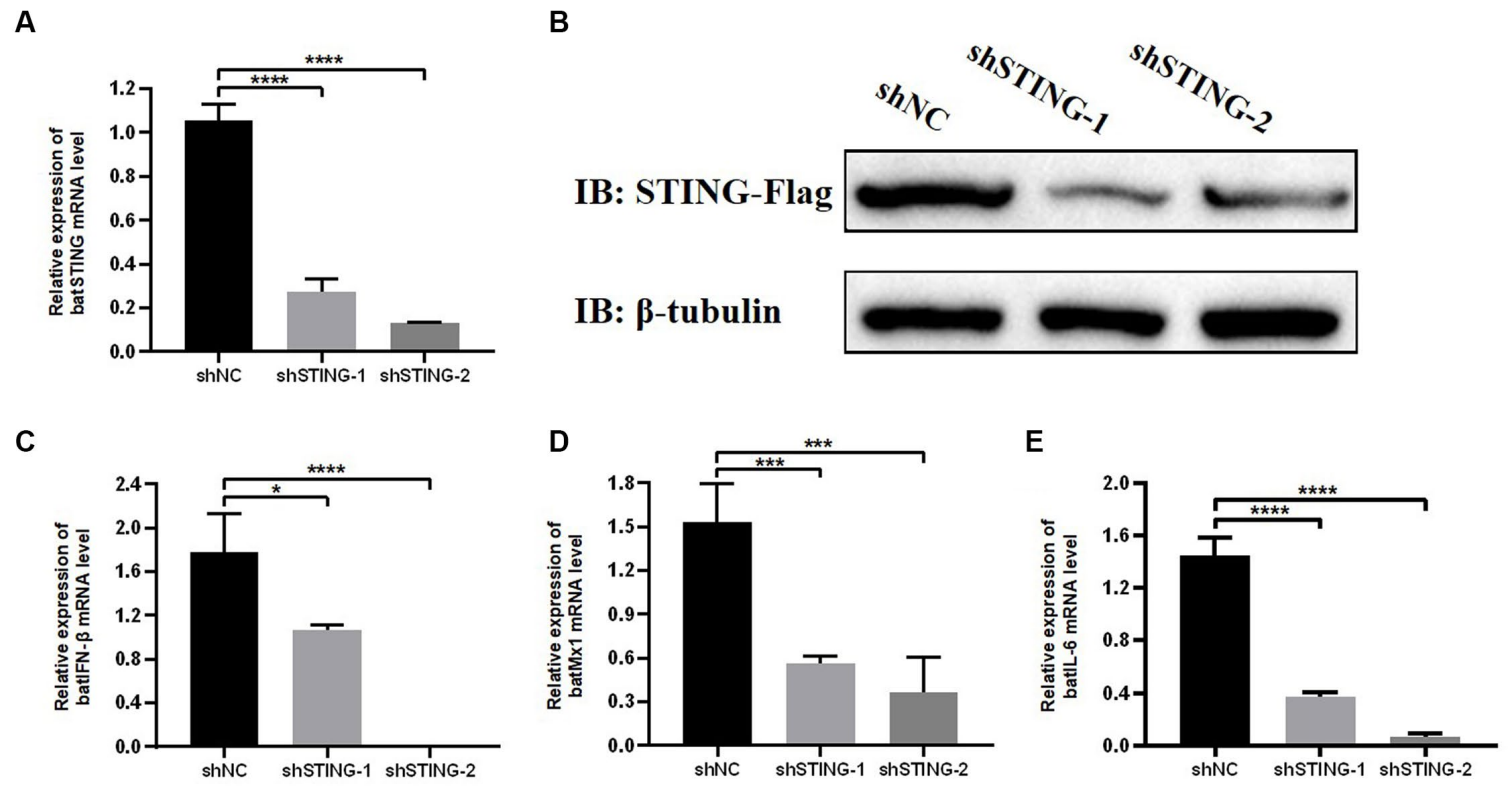
Knockdown of BatSTING blocks IFN-β induction during RNA Virus infection. **(A)** RT-qPCR was used to detect the knockdown efficiency of BatSTING in TB1 Lu cells transfected with shNC or shSTING-1 or shSTING-2, infected with VSV-GFP at 1 MOI for 24 h. **(B)** TB1 Lu cells in 12-well plates overnight were co-transfected with plasmids expressing either C-terminal Flag-tagged BatSTING together with shSTING-1, shSTING-2, or shNC (1 μg). After 24 h, the expression levels of tagged BatSTING and β-tubulin were quantified by western blotting. **(C–E)** RT-qPCR was used to detect the expression level of IFN-β, MX1 and IL-6 in TB1 Lu cells after knockdown of BatSTING, infected with VSV-GFP at 1.0 MOI for 24 h. Data are expressed as the means ± SD of three independent experiments. ^*^p < 0.1; ^***^p < 0.001; ^****^p < 0.0001.

To further investigate the regulatory role of BatSTING on key immune-related genes during antiviral resistance, TB1 Lu cells were transfected with shRNA targeting STING (shSTING-1 or shSTING-2). We then treated wild type (WT) or BatSTING knockdown cells with VSV-GFP infection at 24 h post transfection. To determine the efficiency of the knockdown, total cellular RNA was extracted 24 h after viral infection, and endogenous STING mRNA levels of were quantified by RT-qPCR. The results showed that the endogenous transcription of BatSTING mRNA transcription in TB1 Lu cells was substantially reduced by more than 70% after transfection with the RNAi sequences shSTING-1 and shSTING-2 (Figure 6A). We also further validated the specificity of STING knockdown by Western blot analysis (Figure 6B). It is worth noting that the expression of IFN-β, Mx1 and IL-6 at the mRNA level was significantly down-regulated after knockdown of STING by RNAi plasmid (Figures 6C–E). This implies that BatSTING is endogenously and inextricably linked to the regulation of important antiviral genes and inflammatory factors.

### The essential domains of BatSTING for the activation of the IFN-β

**Figure 7:**
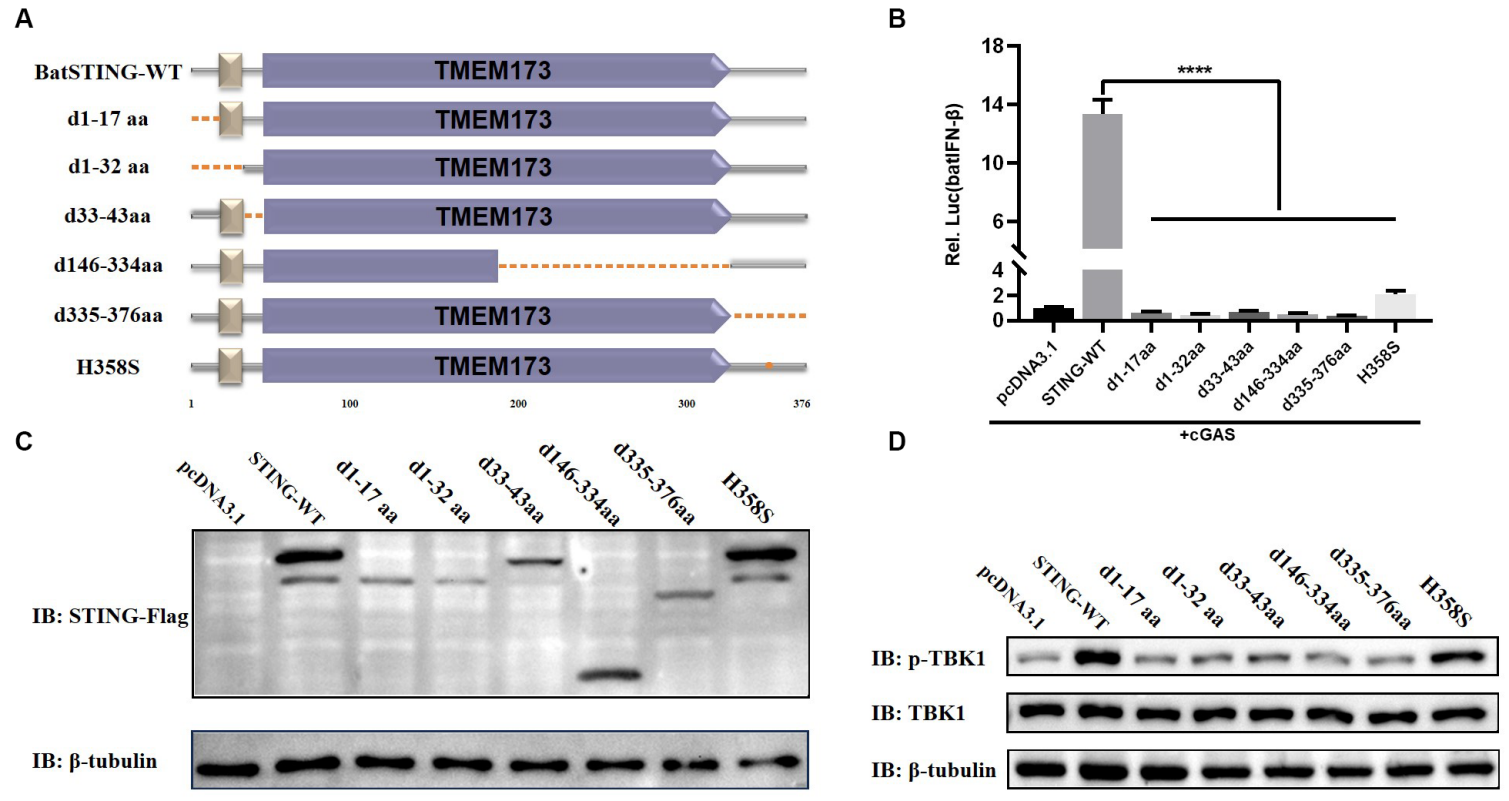
The essential domains of BatSTING for the activation of the IFN-β. **(A)** Schematic structure of BatSTING mutants. **(B)** The effects of BatSTING truncated mutants on IFN-β promoter activity. Cells were transfected with different expression plasmids of BatSTING and the reporter plasmids pGL-batIFN-β-Luc and internal control Renilla luciferase (pRL-TK). Luciferase assays were performed 24 h after transfection. **(C)** Western blots for expression of the truncated BatSTING protein. **(D)** TB1 Lu cells were transfected with mutants of Flag-tagged STING and stimulated with cGAS. Total and phosphorylated TBK1 were detected with TBK1 and p-TBK1 antibodies, respectively. All luciferase assays were repeated at least three times and significance was analyzed with ANOVA (^****^p < 0.0001).

Based on the structural domains of BatSTING predicted by the SMART program, we constructed several mutants lacking multiple functional domains or sites and verified their expression by Western blot (Figures 7A,C). Under conditions of cGAS stimulation, the functional differences in the induction of batIFN-β by various mutants were compared by dual luciferase reporter assays (Figure 7B). Our preliminary analysis shows that the N-terminal deletion mutants (d1-17aa, d1-32aa and d33-43aa), the C-terminal deletion mutant (d335-376aa) and the mutant missing TMEM173 domain fragments (d146-334aa) completely failed to activate the IFN-β promoter at all. Moreover, the mutation of histidine at position 358 (corresponds to the S358 in the human STING) led to such a strong decrease in the ability of BatSTING to activate IFN-β. Alternatively, in order to explore the key regions and sites in STING that affect TBK1 activation, the key steps in the activation and exertion of biological functions of the cGAS-STING signaling pathway, we investigated the effect of STING truncation on TBK1 phosphorylation through Western blot. As shown in Figure 7D, the loss of N-terminal regions and incomplete domains of TMEM173 not only inhibits the synergistic cGAS activation of IFN promoters, but also significantly hinders the phosphorylation of TBK1. Not surprisingly, the deletion of C-terminal amino acids in STING has a serious effect on phosphorylation of TBK1, which is consistent with previous studies that STING mediates TBK1 recruitment and activation through a conserved motif at the C-terminal tail (Zhao et al., 2019). In contrast, after STING’s H358 mutation to serine, the phosphorylation of TBK1 progressed almost normally, indicating that the 358 site of STING is not a key site that affects its binding to TBK1.

## Discussion

Bats are potential reservoirs of zoonotic diseases caused by numerous highly pathogenic RNA viruses, prompting researchers to pay attention to the immune system of bats. Particularly, the ability of bats to keep asymptomatic following viral infections remains a mystery to the scientific fields. It is hypothesized that bats carry these infections without apparent clinical symptoms in part because of the suppression of virus replication in the early stages of their innate immune response (Tarigan et al., 2020). In mammals, the IFN system is one of the most important early antiviral defense systems and plays a key role in host antiviral protection (Zhou et al., 2011). In recent years, it has been increasingly recognized that the IFN response induced by the cGAS-STING pathway is involved in RNA virus infection (Anwar et al., 2021). Nevertheless, due to the wide variety of bats, the role of their immune response in controlling viral infections has not been examined in detail (Baker et al., 2013). Therefore, in this study, we focused on the *in vitro* activity of the BatSTING involved in the production of IFNs with the participation of cGAS using a lung epithelial cell line from the Brazilian free-tailed bat as the research model. Our results clearly indicate that the BatSTING plays an important role in activating IFN-I responses and controlling RNA virus infection, providing further insights into the immunopathogenesis and DNA-sensing cGAS-STING axis-dependent responses of bats during RNA virus infection.

First, we cloned the STING gene of *T. brasiliensis* and performed a series of bioinformatic analyses. The full-length BatSTING gene contains 1,131 bp and encodes 376 amino acids. The prediction results of the secondary structure of BatSTING show that it has a short N-terminal low-complexity region and a typical TMEM173 domain, which has been shown in humans to be essential for retaining STING proteins in the ER or mitochondrial membrane (Zhong et al., 2008; Sun et al., 2009). The analysis of the amino acid sequences of STING from different species show that BatSTING has low homology with other species, with BatSTING protein sequence forming a separate branch in the interspecies phylogenetic tree. The BatSTING shares the highest similarity with STING of cattle. STING of humans and mice share the same 72.3% amino acid identity with BatSTING. What’s more, from a phylogenetic perspective within bat species, STING from Brazilian anurans is most closely related to STING from *M. molossus*. The predicted three-dimensional structure of BatSTING is shown in Figure 2C.

Activation of STING has been found to occur following infection with various RNA viruses (Nazmi et al., 2012). To clarify the relationship between RNA viruses and TB1 Lu cells and the changes of BatSTING expression after infection, we infected TB1 Lu cells with three RNA viruses, NDV-GFP, VSV-GFP and AIV, at a certain dose and examined transcriptional levels of BatSTING at different time points after infection. It was found that BatSTING was significantly activated during RNA virus infection. This suggests that BatSTING is most likely involved in the innate immune response triggered by RNA virus infection. Furthermore, to investigate the interaction between host IFN signaling and RNA viruses, the mRNA expression of several ISGs were measured by RT-qPCR together. The results showed that the expression levels of IFN-β, Mx1, and OAS1 were significantly upregulated by the strong induction of RNA viruses. Although the exact mechanism by which BatSTING is activated still needs further study, it is obvious that BatSTING is required for the host response against RNA viruses.

To further explore the role of STING in regulating RNA virus replication, we overexpressed cGAS and STING separately or together in TB1 Lu cells and infected the cells with VSV-GFP 24 h after transfection. The results showed that the protein expression level of VSV-GFP was significantly reduced in TB1 Lu cells overexpressed jointly by cGAS and STING. This suggests that the cGAS-STING axis can significantly reduce RNA virus production in bat cells during infection. As it is an evolutionarily conserved cytoplasmic DNA recognition pathway of the innate immune system, the main mechanism by which the cGAS-STING axis inhibits RNA virus replication may be the rapid initiation of IFN-dependent immune response activity via the typical cGAS-STING-TBK1 pathway. Thus, we explored the effect of cGAS-STING signaling on the activation of batIFN-β. Unsurprisingly, The joint overexpression of cGAS and STING activates batIFN-β promoters by a factor of about 10. Compared to the strong induction of IFN-β in humans and mice, the IFN-β activation response induced by BatSTING is relatively mild. After IFN-β production, it binds to specific receptors to further induce the expression of ISGs. Among these ISGs, Mx1, PKR and OAS1 represent the most predominant antiviral pathway and are the most widely studied in bats so far (Zhou et al., 2013). RT-qPCR results showed that Mx1, PKR and OAS1 in TB1 Lu cells were all significantly induced to be upregulated by overexpression of STING alone or co-expression with cGAS (Figures 5C–E). In addition, STING significantly activated the expression of several pro-inflammatory cytokines including IL-6, TNF-α, and IL-1β (Figures 5F–H).

It should be mentioned that the cGAS gene used in this study is cloned from human cells. Although we have done our best to clone cGAS from bats, unfortunately, it has not been successful. This may due to the lack of cGAS sequences from *T. brasiliensis* in the GenBank database, the poor conservatism of cGAS genes in species, as well as the low basal expression of immune genes in bat cells. To compensate for the shortcomings caused by the substitution of a heterologous cGAS and investigate whether the involvement of BatSTING is indeed necessary for the type I IFN response elicited by RNA viruses and, we use RNAi experiments to analyze the effect of STING knockdown on the IFN response in TB1 Lu cells. As shown in Figure 6A, the level of endogenous STING expression was significantly decreased in TB1 Lu cells transfected with the RNAi plasmid. Western blot results also showed the success of our construction of STING-deficient TB1 Lu cell lines. Immediately after transfection for 24 h, we infected TB1 Lu cells with VSV-GFP. The expression levels of IFN-β, Mx1 and IL-6 were significantly down-regulated by RT-qPCR assay 24 h after virus stimulation, implying that BatSTING functions at the corresponding endogenous levels in its native species. The above results suggest that knockdown of STING prevents the activation of IFN-β and that STING is necessary for the induction of IFN-β as well as other critical antiviral factors by the host during RNA virus infection.

To identify the key domains and sites that induce TBK1 activation and IFN-β production in BatSTING, we constructed a series of truncated forms of BatSTING mutants and examined their effect on TBK1 phosphorylation by Western blot and their ability to activate the batIFN-β promoters by a dual luciferase reporter assay. The results showed that in the deletion of amino acid fragments of TMEM173 domain significantly inhibited the phosphorylation of TBK1 and eliminated the ability of BatSTING to activate IFN-β, indicating the importance of the TMEM173 domain. It has been reported that STING recruits and activates TBK1 through the carboxyl terminus to phosphorylate IRF3, and phosphorylated IRF-3 is dimerized subsequently into the nucleus to initiate IFN-β expression (Ishikawa and Barber, 2008). This is consistent with our results that BatSTING mutants lacking 42 amino acids at C-terminal (BatSTING-d335-376aa) have significantly reduced ability to activate the IFN-β promoter, with TBK1 phosphorylation showing the same trend. In addition, BatSTING-d1-17aa, BatSTING-d1-32aa, and BatSTING-33-43aa, with deletion of only 17, 32, and 10 amino acids at the N-terminal of BatSTING, respectively, also led to a strong decrease in TBK1 and IFN-β induction, which may be related to their problematic intracellular localization. Zhou et al. found that the inhibition of the IFN response in bats was due to the substitution of a highly conserved serine residue (S358) in STING (Xie et al., 2018). To verify whether amino acid at position 358 has the same effect on STING’s ability to activate IFN in TB1 Lu cells, we mutated the histidine at position 358 in BatSTING to serine and examined the ability of the mutant to activate IFN-β. The results showed that the ability of BatSTING-H358S to activate IFN-β was significantly down-regulated compared to WT BatSTING, implying that the amino acid at position 358 of BatSTING does not inhibit the ability of IFN activation, but rather has a positive effect on IFN-β induction. In addition, phosphorylation of TBK1 proceeded almost normally with the mutation of H358 of STING to serine, suggesting that the 358 site of STING is not a critical site affecting its function.

To sum up, this study systematically investigated the role of BatSTING in regulating the innate immune response and coping with RNA virus infection. The study found that with the participation of cGAS, the overexpression of BatSTING could moderately increase the transcription of IFN-β and some ISGs, and effectively inhibit the replication of VSV-GFP in TB1 Lu cells, suggesting that BatSTING is an important limiting factor for RNA virus infection. This study provides fundamental and novel insights into innate immunity in bats and has implications for exploring the mechanisms controlling bat virus replication and explaining the pathogenicity of RNA viruses to humans and other hosts.

## Data availability statement

The raw data supporting the conclusions of this article will be made available by the authors, without undue reservation.

## Author contributions

YC and JS designed the experiment. FF and QS performed the majority of the experiments. JZ and JW helped with the experiments. FF and YC wrote the paper. ZW, JM, and YY helped analyze the experimental results. All authors contributed to the article and approved the submitted version.

## Funding

This research was supported by the National Natural Science Foundation of China (32072865 and 32072864), the Natural Science Foundation of Shanghai (20ZR1425100), the Open Project Program of Jiangsu Key Laboratory of Zoonosis (No. R2102), and State Key Laboratory of Veterinary Biotechnology Foundation Grant (SKLVBF202107).

## Conflict of interest

The authors declare that the research was conducted in the absence of any commercial or financial relationships that could be construed as a potential conflict of interest.

## Publisher’s note

All claims expressed in this article are solely those of the authors and do not necessarily represent those of their affiliated organizations, or those of the publisher, the editors and the reviewers. Any product that may be evaluated in this article, or claim that may be made by its manufacturer, is not guaranteed or endorsed by the publisher.

## Footnotes

^1^http://smart.embl-heidelberg.de/

^2^http://http://espript.ibcp.fr/ESPript/cgi-bin/ESPript.cgi

^3^http://swissmodel.expasy.org/

^4^https://www.genscript.com/ssl-bin/app/rnai
